# Comparison of Qualitative (Time Intensity Curve Analysis), Semi-Quantitative, and Quantitative Multi-Phase 3T DCE-MRI Parameters as Predictors of Malignancy in Adnexal

**DOI:** 10.31557/APJCP.2019.20.6.1603

**Published:** 2019

**Authors:** Mahrooz Malek, Zeinab Oghabian, Elnaz Tabibian, Maryam Rahmani, Seyedeh Nooshin Miratashi Yazdi, Mohammad Ali Oghabian, Sara Parviz

**Affiliations:** 1 *Advanced Diagnostic and Interventional Radiology Research Center (ADIR), *; 2 *Neuroimaging and Analysis Group, Research Center for Molecular and Cellular Imaging, *; 3 *Department of Radiology, Medical Imaging Center, Imam Khomeini Hospital, Tehran University of Medical Sciences, Tehran, Iran. *

**Keywords:** Adnexal mass, DCE-MRI, qualitative, semi-quantitative, quantitative

## Abstract

**Objective:**

The present study aimed to compare the qualitative (time intensity curve analysis), the semi-quantitative and the quantitative multiphase 3T dynamic contrast-enhanced (DCE) MRI parameters as predictors of malignancy in adnexal masses. **Materials and **

**Methods:**

In this prospective study, women with an adnexal mass who were scheduled for surgical resection or were followed for more than one year period to confirm the benignity of their lesions, underwent multiphase 3T DCE-MRI. The qualitative (time intensity curve), semi-quantitative (SImax, SIrel, WIR) and quantitative (Ktrans, Kep, Vb) analyses were performed on DCE-MRI sequences and their predictive values were compared.

**Results:**

**A** total of 17 benign and 14 malignant lesions were included. According to the qualitative analysis, none of the lesions with Type I time intensity curves (TIC) were malignant and none of the masses with Type III TICs were benign. The accuracy of the quantitative parameters in detection of malignancy was found to be higher than that of semi-quantitative variables, particularly when calculated for a small ROI within the high signal area of the mass (sROI) rather than the largest ROI including the whole mass (lROI), and when inter-MRI variations were omitted using ratios. The Kep(tumor)/Kep(myometrium) ratio measured from sROI was the best parameter for differentiating a malignant lesion with a sensitivity of 100% and a specificity of 92.3%.

**Conclusion:**

We concluded that a Type I TIC confirms a benign lesion, and a type III TIC confirms the malignancy and further evaluation is not recommended for these lesions. So complementary quantitative analysis is only recommended for adnexal masses with type II TICs.

## Introduction

Differentiating malignant adnexal masses from the benign ones is an important issue in gynecology, since the management (medical or surgical), type of surgery and prognosis of the patients depend on the benignity or malignancy of the lesions (Siegel et al., 2016). Routinely, ultrasound assessment is used for the characterization of adnexal masses. However, up to 20% of such lesions identified by ultrasound cannot be determined via this method (Imaoka et al., 2006; Bent et al., 2006). In these cases, contrast-enhanced MRI is now widely accepted as the next best step for the characterization of the mass and has been shown to offer a high specificity and sensitivity for differentiating among benign and malignant lesions (Kuhl et al., 2000; Sohaib et al., 2003; Bazot et al., 2006) 

Even after using this modality, some adnexal masses still remain indeterminate (Imaoka et al., 2006), for which diffusion weighted MRI (DWI) has been suggested as the solution, but enough evidence was not available to support the fact that DWI could independently characterize an adnexal mass (Torbati et al., 2014; Thomassin-Naggara et al., 2005). Accordingly, Thomassin Naggara et al. proposed the application of dynamic contrast-enhanced MRI (DCE-MRI), in which tissue enhancement is evaluated according to the time. This method is more accurate than conventional contrast enhanced MRI and could differentiate among the benign and malignant lesions on the basis of the differences in their enhancement parameters (Thomassin Naggara et al., 2008). 

Three separate qualitative, semi-quantitative and quantitative parameters can be used in DCE-MRI to gather different types of information from the imaging procedure. For the qualitative assessment, a time-intensity curve (TIC) is drawn and according to the temporal changes of the curve, conclusions are made regarding the risk of malignancy (Kuhl et al., 1999). This method is user-friendly, and TIC could be drawn by the radiologist on the MRI machine workstation and does not need any complicated mathematical assessments by a physicist. As for the semi-quantitative evaluation, the parameters are calculated by Matlab software based on the time intensity curve, including the maximum signal intensity (SImax), the maximum signal intensity relative to the tissue signal intensity on the non-enhanced T1-weighted sequence (SIrel), the wash-in rate (WIR) and the area under the curve of the first 60 seconds in the time-intensity curve (AUC-60).

In the quantitative assessment, the transfer constant (Ktrans), rate constant (Kep), blood volume (Vb), and extravascular extracellular volume fraction (Ve) were calculated using a pharmacokinetic model and microcirculation parameters on the basis of a higher neoangiogenesis and vascular permeability in malignant lesions.

Previous surveys have assessed the effectiveness of DCE-MRI in differentiating among the malignant and benign lesions of various tumors such as prostate, breast and ovaries (Kuhl et al., 2000; Ocak et al., 2007; Bernardin et al., 2012). The three different qualitative, semi-quantitative, and quantitative methods of DCE-MRI have also been compared for prostate and breast cancers, but no such studies are available for adnexal masses. Accordingly, we aimed to compare the diagnostic value of multiple qualitative, semi-quantitative, and quantitative dynamic contrast-enhanced MRI parameters in this study. 

## Materials and Methods

In this prospective study, the diagnostic value of different qualitative, semi-quantitative and quantitative parameters of DCE-MRI in the characterization of adnexal masses was evaluated. The study protocol was approved by the ethics committee. Informed written consent was obtained from all patients who were willing to participate in the study and all the gathered data were considered confidential. 

*Patients*

All the women who were scheduled for adnexal mass surgery or given at least one year’s follow- up period to confirm the benignity of their lesion had been evaluated between March, 2014 and June, 2015. Patients who met the inclusion and exclusion criteria of the survey were included as the study population. 

*The inclusion criteria were as follows:*

Pathology-proven adnexal mass with surgery (laparoscopic or open surgery) or at least a one-year follow up to confirm a benign diagnosis

Underwent DCE-MRI with acceptable quality images (lacks image artifacts or other technical issues)

Presence of solid enhancing components on the tumor that are at least 3mm in diameter.

*The exclusion criteria included:*

Any reason that prohibits the patients from undergoing DCE-MRI such as claustrophobia, inability to lie still for 30-60 minutes, weighing over 120 kg, having an implant, cardiac pacemaker, insulin pump or cochlear implant, fixator of the spine, brain aneurysm clips, pregnancy, severe respiratory disorder or any contraindication for contrast injection like GFR under 30ml/min

No pathology report or an indistinctive diagnosis in the report or a follow-up period of less than one year to confirm a benign lesion.

*Not willing to participate*

*Imaging Protocol*

Magnetic resonance imaging was performed using 3-Tesla MR (Siemens, MAGNTOM Trio, Avanto, phased array coil surface) in 31 patients. After fasting for 3 hours, the patients received 20mg of Hyoscine Butylbromide intramuscularly to induce anti-peristaltic effects right before the MR imaging. Conventional MRI of the pelvic region was obtained through the routine protocol. The range of scan was set between the umbilicus and the pubic symphysis. Next, based on the findings of initial non-enhanced sequences, DCE-MRI was performed from the tumor’s solid components (papillary projection, solid nodule, or thickened septa), using 3D VIBE sequences of 45 measurements with 5s/frame temporal resolution. Image acquisition was carried out before and immediately after a single dose of the 0.2 mL/kg gadolinium (Dotarem; Guerbet, Aulnay, France) injection at a rate of 3 mL/min, followed by 20cc of normal saline.

*Qualitative analysis*

For qualitative assessment, several regions of interest (ROI) with the largest possible ROI and pinpointed ROI were placed on the solid component of the adnexal masses or on their thick walls or septae (in adnexal masses without solid component) by a radiologist with six years of experience in gynecologic-oncology imaging, blinded to the proven diagnosis of the patients. The TIC was also drawn for the psoas muscle and myometrium. The highest curve type for each adnexal mass was selected for analysis. 

The enhancement of kinetic curves or TICs were produced for the lesions by Syngo software on the Siemens workstation (Siemens, Germany). According to the temporal changes, the curves were categorized into three types ( Kuhl et al., 1999;Thomassin-Naggara et al., 2015): Type I was defined as a mildly persistent gradual increase without a shoulder, Type II as a moderate initial increase followed by a plateau and Type III as a relatively rapid uptake followed by reduction in enhancement towards the latter part of the study. 

*Semi-quantitative analysis*

The same radiologist, analyzed the multiphase dynamic images obtained. In the semi-quantitative and quantitative assessments, two types of ROIs were drawn over the adnexal masses. One outlined the whole circumference of the solid part of the lesion in the section with maximum area of the tumor, referred to as the large ROI (lROI). For the second ROI, referred to as the small ROI (sROI), the solid component of the lesion with the highest enhancement was identified and a circular ROI with a diameter of greater than 3 mm was manually drawn.

sROIs were drawn for the psoas muscle and myometrium as two internal references as well. A single high signal point was also chosen in all the three ROIs of the tumor, psoas muscle and myometrium. The semi-quantitative analysis was performed using the Matlab software ( Ji et al., 2007) . The following parameters were calculated automatically: 

SI_max_: maximum absolute enhancement

SI_rel_: maximum enhancement relative to the tissue signal intensity on non-enhanced T1-weighted sequence (SI_0_)

${\mathrm{SI}}_{\mathrm{rel}}=\frac{{\mathrm{SI}}_{\max}-{\mathrm{SI}}_{0}}{{\mathrm{SI}}_{0}}\times 100$

WIR: wash-in rate

$\mathrm{WIR}=\frac{{\mathrm{SI}}_{0}-{\mathrm{SI}}_{\max}}{\mathrm{time}(S)}$

AUC-60: the area under the curve for the first 60 seconds in the time-intensity curve 

To reduce the inter-MRI variations, the ratios of these parameters calculated for the tumor relative to that of the psoas muscle and myometrium were also calculated for both lROI and sROI as well as the selected high-signal points in each patient. 

*Quantitative analysis*

In the quantitative assessment, the transfer constant (K_trans_), rate constant (K_ep_), blood volume (Vb) and the extravascular extracellular volume fraction (Ve) were calculated using a pharmacokinetic model. The concentration of the contrast in the plasma was calculated through an arterial input function (AIF) model. The population-based AIF was calibrated to the AIF obtained from 10 randomly selected patients among our study population in whom the iliac artery was selected as the region of the arterial input. The pharmacokinetic parameters were calculated in accordance with the following formulae:

$K_{\mathrm{ep}}=\frac{1}{{\mathrm{ttp}}_{\mathrm{tissue}}-{\mathrm{ttp}}_{\mathrm{plasma}}}$

$K_{\mathrm{trans}}=V_{e}\times K_{\mathrm{ep}}$

$V_{e}=\frac{C_{\mathrm{gdtissue}}}{C_{\mathrm{gdplasma}}}$

where C_gdplasma_ and C_gdtissue_ are the concentrations of contrast in plasma and tissue respectively, and ttp_plasma _and ttp_tissue_ are the times to reach the peak intensity in plasma and tissue, respectively. 

Similar to the method applied for semi-quantitative analyses, the mentioned parameters were established for the psoas muscle and myometrium as well, and their corresponding ratios were also calculated for both lROI and sROI. 

*Statistical analysis*

The SPSS software for windows, version 22, was used for statistical analysis (SPSS, Chicago, IL, USA, 2015). The differences in DCE-MRI variables between the two groups of benign and malignant lesions were evaluated via the independent samples t-test. The Receiver Operating Characteristic (ROC) Curve analysis was used to assess and compare the diagnostic values of the measured parameters based on the area under the ROC curves. The best cut-off values were chosen on the basis of the coordinate points of the ROC curves, and their sensitivity, specificity, positive predictive value (PPV), and negative predictive value (NPV) were calculated accordingly. A P-value of less than 0.05 was considered as statistically significant in all analyses. 

## Results

Data gathered from the 31 patients was included in the analyses,the histopathology findings of which are presented in Table 1. As it can be seen, 17 patients had benign lesions and the remaining 14 subjects were diagnosed with malignancies. 

*Qualitative analysis*

Based on the results of the qualitative analysis, all nine patients with Type I curves were found to have benign lesions, while all the ten subjects with Type III curves had malignant tumors. Among the 12 patients with Type II curves, eight (66.7%) had benign lesions and four (33.3%) had malignant ones (p<0.001). To calculate the sensitivity, specificity, PPV, and NPV of the Type I curve for diagnosing benign lesions, Type II and III curves were grouped together. The results showed a sensitivity of 53%, specificity of 100%, PPV of 100%, and NPV of 63.6%. Similarly, to calculate these parameters of the Type III curve in diagnosing malignant lesions, Type I and II curves were grouped together and the results yielded a sensitivity of 71.4%, specificity of 100%, PPV of 100%, and NPV of 80.9%. 

*Semi-quantitative analysis*

Table 2 presents the results of the semi-quantitative analysis on the data. From the evaluations performed on lROI measurements, SI_rel_ (p=0.021) and WIR (p=0.046) were found to be significantly higher in patients with malignant lesions. SI_rel_(tumor)/SI_rel_(psoas) (p=0.001) and SI_rel_(tumor)/SIrel(myometrium) (p=0.031) were also found to be significantly higher in subjects with malignant tumors. The assessments on the sROIs also showed that SI_rel_(tumor)/SI_rel_(myometrium) (p=0.009) and WIR(tumor)/WIR(myometrium) (p=0.006) were significantly higher in malignant lesions. 

*Quantitative analysis*

The results of the quantitative analyses are presented in Table 3. As illustrated, most of the evaluated variables are found to be significantly higher among the patients with malignant lesions. The only parameters where their differences had been found to be insignificant were the V_e_ of both sROI and lROI (p=0.526 and p=0.117 respectively) and the V_b_(tumor)/V_b_(psoas) (p=0.335) of the lROIs.

*Diagnostic value of the parameters*

**Figure 1:**
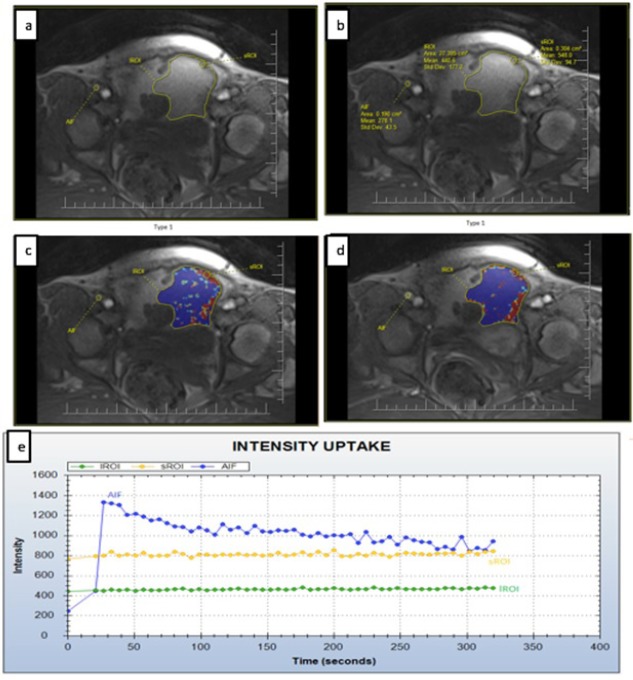
Type I Intensity Curve Along with the Corresponding Quantitative Measurements on the Patient’s MRI; (a) Source; (b) Details of the source; (c) Kep; (d) Ktrans; (e) TIC

**Figure 2:**
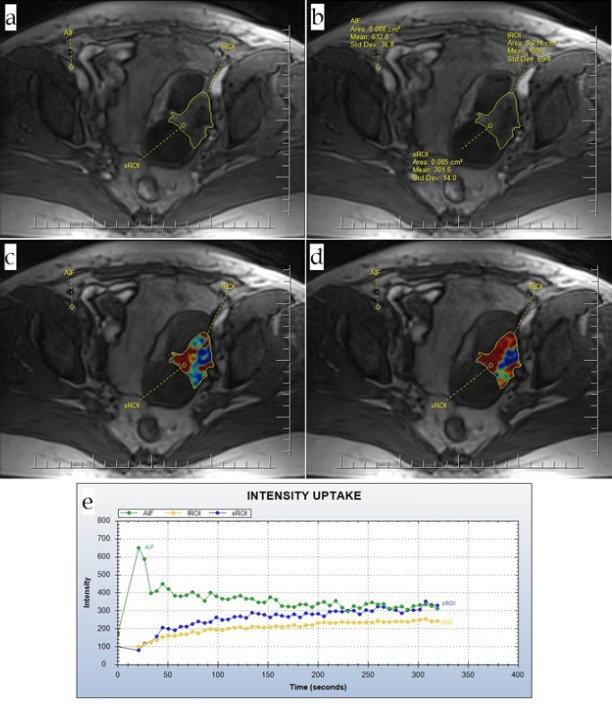
Type II Intensity Curve Along with the Corresponding Quantitative Measurements on the Patient’s MRI; (a) Source; (b) Details of the source; (c) Kep; (d) Ktrans; (e) TIC

To assess the diagnostic value of the evaluated factors in the study, the AUC of ROC curves were calculated for each parameter. As presented in Table 4, the highest AUCs of lROI parameters were measured for K_trans_(tumor)/K_trans_(myometrium), AUC-60(tumor)/AUC-60 (myometrium), and V_b_(tumor)/V_b_(myometrium). The highest AUCs of parameters assessed on sROI were found in K_ep_(tumor)/ K_ep_ (myometrium), K_trans_(tumor)/K_trans_(myometrium), V_b_(tumor)/V_b_(myometrium), and AUC-60(tumor)/AUC-60(myometrium). The ROC curves of these parameters with the highest AUCs are depicted in Figures 1 and 2, according to which the best cut-off value, sensitivity, specificity, PPV, and NPV were calculated. As presented in Table 5 the K_ep_(tumor)/K_ep_(myometrium) ratio measured from sROI within the outlined ROI is the best parameter for differentiating a malignant lesion with a sensitivity of 100%, specificity of 92.3%, PPV of 91.4%, and NPV of 100%. 

**Figure 3:**
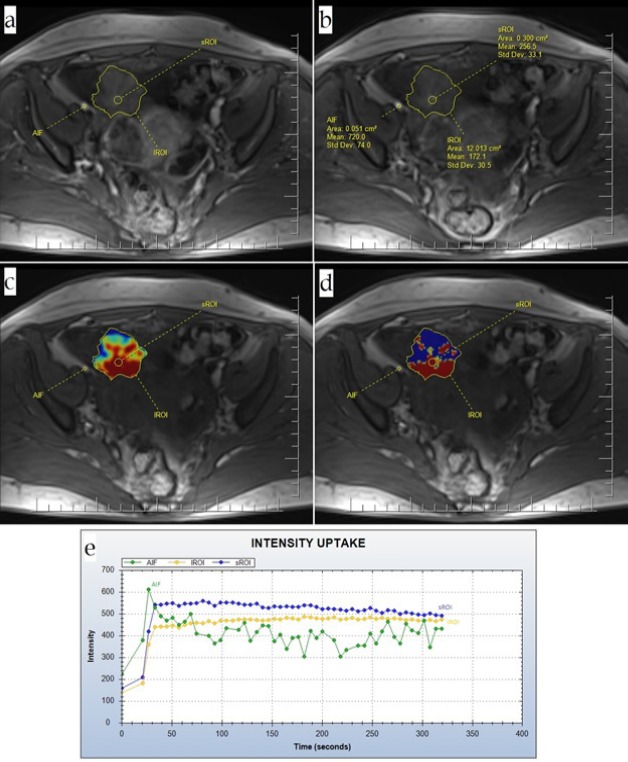
Type III Intensity Curve Along with the Corresponding Quantitative Measurements on the Patient’s MRI; (a) Source; (b) Details of the source; (c) Kep; (d) Ktrans; (e) TIC

**Figure 4:**
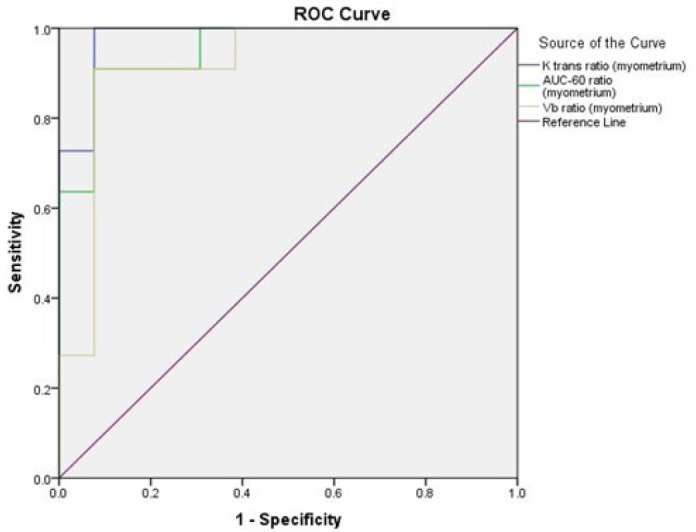
ROC Curves of the Three lROI Measured Parameters with the Highest AUCs

**Figure 5:**
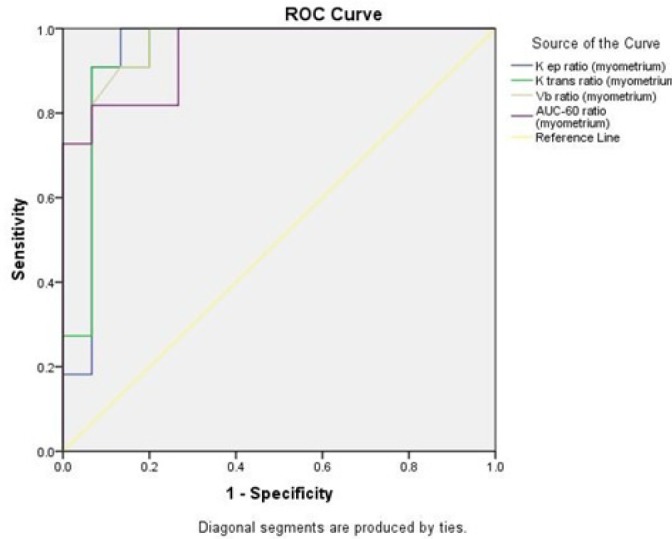
ROC curves of the three sROI measured parameters with the highest AUCs

**Table 1:** Histopathology Results of the Sample Population

Group	Histopathology	Number
Benign (n=17)	Bilateral endometriosis	2
	Endometrioid cystadenoma	1
	Endometrioma	3
	Fibrothecoma	2
	Hemorrhagic cyst	1
	Mature cystic teratoma	2
	Mucinous cystadenoma	1
	Peritoneal inclusion	1
	Serous cystadenofibroma	2
	Serous cystadenoma	1
	TB	1
	Non specified	1
Malignant (n=14)	Choriocarcinoma	1
	Dysgerminoma	1
	Leiomyosarcoma	1
	Metastases	2
	Mucinus cystadenocarcinoma	1
	Papillary Serous cystadenocarcinoma	3
	Serous cystadenocarcinoma	5
Total		31

**Table 2:** Semi-Quantitative Analyses

Region	Parameter	Malignancy		P value
	Mean (Standard Deviation)	Benign	Malignant	
lROI	SI max	274.8 (159.8)	289.2 (87.1)	0.765
	SI rel	53.7 (55.6)	95.7 (35.2)	0.021
	WIR	0.68 (0.6)	1.3 (1.2)	0.046
	AUC-60	3.7 (5.0)	12.3 (6.7)	<0.001
	SI max ratio (psoas)	1.5 (0.8)	1.9 (0.4)	0.17
	SI rel ratio (psoas)	1.3 (1.9)	4.3 (2.6)	0.001
	WIR ratio (psoas)	1.3 (1.8)	4.6 (8.8)	0.151
	AUC-60 ratio (psoas)	1.1 (1.7)	4.3 (3.8)	0.004
	SI max ratio (myometrium)	0.9 (0.5)	1.0 (0.3)	0.531
	SI rel ratio (myometrium)	0.4 (0.6)	1.0 (0.8)	0.033
	WIR ratio (myometrium)	0.4 (0.5)	0.9 (0.5)	0.051
	AUC-60 ratio (myometrium)	0.1 (0.2)	1.2 (1.0)	0.001
sROI	SI max	351.1 (199.2)	361.6 (105.9)	0.861
	SI rel	77.2 (90.4)	106.2 (30.4)	0.262
	WIR	1.0 (0.8)	3.8 (7.4)	0.141
	AUC-60	4.5 (5.8)	12.8 (6.5)	0.001
	SI max ratio (psoas)	2.0 (1.1)	2.4 (0.6)	0.231
	SI rel ratio (psoas)	1.9 (2.7)	4.6 (2.5)	0.009
	WIR ratio (psoas)	2.1 (2.6)	8.9 (14.2)	0.098
	AUC-60 ratio (psoas)	1.3 (1.8)	4.7 (3.6)	0.004
	SI max ratio (myometrium)	1.0 (0.5)	1.2 (0.4)	0.244
	SI rel ratio (myometrium)	0.4 (0.5)	1.0 (0.5)	0.009
	WIR ratio (myometrium)	0.5 (0.7)	1.7 (1.3)	0.006

**Table 3:** Quantitative Analyses

Region	Parameter	Malignancy		P value
	Mean (Standard Deviation)	Benign	Malignant	
lROI	K trans	0.3 (0.3)	0.5 (0.2)	0.015
	K ep	0.4 (0.2)	0.6 (0.2)	0.036
	Vb	0.2 (0.2)	0.6 (0.4)	<0.001
	Ve	0. 7 (0.4)	0.9 (0.2)	0.117
	K trans ratio (psoas)	0. 9 (1.0)	3.3 (2.9)	0.003
	K ep ratio (psoas)	0. 8 (0.6)	1.3 (0.7)	0.028
	Vb ratio (psoas)	3255.4 (12341.3)	12.5 (24.5)	0.335
	Ve ratio (psoas)	1.0 (0.9)	2.5 (1.8)	0.004
	K trans ratio (myometrium)	0.4 (0.4)	1.4 (1.1)	0.003
	K ep ratio (myometrium)	0.5 (0.4)	1.0 (0.3)	0.006
	Vb ratio (myometrium)	0.2 (0.3)	1.3 (1.0)	0.002
	Ve ratio (myometrium)	0.7 (0.5)	1.7 (1.8)	0.05
	K trans	0.3 (0.3)	0.5 (0.2)	0.015
	K ep	0.4 (0.2)	0.6 (0.2)	0.036
	Vb	0.2 (0.2)	0.6 (0.4)	<0.001
sROI	K trans	0.3 (0.3)	0.5 (0.2)	0.034
	K ep	0.4 (0.3)	0.6 (0.2)	0.018
	Vb	0.2 (0.3)	0.6 (0.3)	0.002
	Ve	0.8 (0.4)	0.8 (0.3)	0.526
	K trans ratio (psoas)	1.1 (1.3)	3.6 (3.2)	0.014
	K ep ratio (psoas)	0.8 (0.7)	1.5 (0.7)	0.024
	Vb ratio (psoas)	4238. 3 (14458.2)	12.5 (20.0)	0.246
	Ve ratio (psoas)	1.3 (1.3)	2.1 (1.2)	0.059
	K trans ratio (myometrium)	0.4 (0.4)	1.4 (0.5)	<0.001
	K ep ratio (myometrium)	0.5 (0.4)	1.1 (0.3)	<0.001
	Vb ratio (myometrium)	0.2 (0.3)	1.4 (1.2)	0.001
	Ve ratio (myometrium)	0.7 (0.4)	1.2 (0.5)	0.018

**Table 4:** Area Under the Curve (AUC) for Semi-Quantitative and Quantitative Parameters

Analysis	Region	Test Result Variable(s)	Area	Std. Error	P value
		Smax	0.528	0.124	0.817
Semi- quantitative	lROI	SI rel	0.745	0.108	0.043
		WIR	0.731	0.104	0.056
		SI max ratio (psoas)	0.71	0.115	0.082
		SI rel ratio (psoas)	0.871	0.074	0.002
		WIR ratio (psoas)	0.724	0.107	0.064
		SI max ratio (myometrium)	0.626	0.116	0.297
		SI rel ratio (myometrium)	0.829	0.084	0.006
		WIR ratio (myometrium)	0.843	0.085	0.005
		Smax	0.598	0.12	0.417
	sROI	SI rel	0.738	0.103	0.049
		WIR	0.745	0.114	0.043
		SI max ratio (myometrium)	0.682	0.113	0.132
		SI rel ratio (myometrium)	0.794	0.096	0.015
		WIR ratio (myometrium)	0.864	0.077	0.003
		Ktrans	0.86	0.076	0.003
Quantitative	lROI	Kep	0.776	0.096	0.022
		Vb	0.892	0.064	0.001
		AUC60	0.86	0.081	0.003
		Ve	0.685	0.11	0.125
		K trans ratio (psoas)	0.881	0.068	0.002
		K ep ratio (psoas)	0.79	0.092	0.016
		Vb ratio (psoas)	0.738	0.108	0.049
		AUC-60 ratio (psoas)	0.825	0.086	0.007
		Ve ratio (psoas)	0.839	0.08	0.005
		K trans ratio (myometrium)	0.979	0.025	<0.001
		K ep ratio (myometrium)	0.888	0.066	0.001
		Vb ratio (myometrium)	0.916	0.063	0.001
		AUC-60 ratio (myometrium)	0.951	0.041	<0.001
		Ve ratio (myometrium)	0.727	0.103	0.06
	sROI	Ktrans	0.846	0.08	0.004
		Kep	0.829	0.083	0.006
		Vb	0.888	0.066	0.001
		AUC60	0.888	0.074	0.001
		Ve	0.605	0.117	0.385
		K trans ratio (myometrium)	0.986	0.018	<0.001
		K ep ratio (myometrium)	0.993	0.011	<0.001
		Vb ratio (myometrium)	0.972	0.028	<0.001
		AUC-60 ratio (myometrium)	0.937	0.047	<0.001
		Ve ratio (myometrium)	0.755	0.098	0.034

**Table 5:** Cut-off Value, Sensitivity, Specificity, PPV and NPV of the Best Diagnostic Parameters

Region	Test Result Variable(s)	Area	Cut-off	Sensitivity	Specificity	PPV	NPV
lROI	K trans ratio (myometrium)	0.979	0.53	100	92.3	91.4	100
	AUC-60 ratio (myometrium)	0.951	0.46	90.9	92.3	90.6	92.5
	Vb ratio (myometrium)	0.916	0.4	90.9	92.3	90.6	92.5
sROI	K ep ratio (myometrium)	0.993	0.73	100	92.3	91.4	100
	K trans ratio (myometrium)	0.986	0.82	90.9	100	100	93
	Vb ratio (myometrium)	0.972	0.49	90.9	92.3	90.6	92.5
	AUC-60 ratio (myometrium)	0.937	0.44	81.8	92.3	89.7	86.1

## Discussion

Differentiating among malignant adnexal masses and benign lesions is of utmost importance to determine the best course of action for the patient. Despite the vast improvements in imaging techniques and modalities, a few cases still remain indeterminate (Hricak et al., 2000). Therefore, better diagnostic tools and criteria are needed to enhance our ability to differentiate an invasive lesion from a benign one. In this regard, the superiority of DCE sequences compared with simple CE sequences for qualitative evaluation of enhancement has been established (Sohaib et al., 2003; Husband et al., 2006;Thomassin-Naggara et al., 2008; 2011). Moreover, three different analyses have been proposed to acquire further information from the DCE-MRI that might be valuable in distinguishing malignant lesions from the benign ones. Qualitative analysis categorizes the patients into three groups based on the temporal changes of enhancement, observed in the kinetic curves. The method was described by Kuhl et al., (1999), and its effectiveness was evaluated in distinction of malignant and benign breast masses. They reported a sensitivity of 91%, specificity of 83%, and a diagnostic accuracy of 86% for the Type III curves in diagnosing malignant lesion and concluded that the type III time-course is a strong predictor of malignancy. Qualitative analysis is a user-friendly and simple method compared with other mentioned methods for DCE-MRI assessment. According to the results of the present survey, an adnexal mass is benign if it presents a Type I TIC, and the mass is malignant if it presents Type III TIC, and therefore, there is no need for application of semi-quantitative and quantitative methods in these conditions.

Semi-quantitative analysis derives multiple parameters from the TICs drawn for the lesion. Bernardin et al., (2011) assessed the diagnostic value of this method in predicting malignancy in adnexal masses; they found significant differences in SImax, SIrel, and WIR between malignant and benign lesions. A cut-off WIR of equal or greater than 9.5 was found to have a specificity of 88% and PPV of 86%. In our previous study in 2014, in order to reduce inter-MRI variation we included ratios of these parameters in the lesion to that of psoas and myometrium and reached promising results (Torbati et al., 2014). WIR, AUC-60, SI_max_(tumor)/SI_max_(psoas), SI_max_(tumor)/SI_max_(myometrium), WIR(tumor)/WIR(psoas) and WIR(tumor)/WIR(myometrium) were significantly higher in malignant tumors. 

Quantitative analysis is based on the higher neoangiogenesis and vascular permeability in malignant lesions ; it uses a pharmacokinetic model and microcirculation parameters. Priest et al., (2010) aimed to compare these variables between malignant and benign ovarian masses and found no significant difference between the two groups by considering K_trans_, K_ep_, V_b_, V_e_, and the AUC of the first 60 seconds in the TIC. However, Amaranth and colleagues (2013) conducted an ROC curve analysis and showed that a mean K_trans_ value of 0.56 reliably differentiates malignant lesions from benign masses with a sensitivity of 91.1%, a specificity of 90.3%, and an overall accuracy of 89.3%. 

In this regard we aimed to compare the efficacy of these three analyses while adding some ratio parameters to the model in order to reduce inter-MRI variation (Torbati et al., 2014). Other than performing these analyses on the outlined ROIs of the tumor, psoas muscle and myometrium, we also measured these factors for a small ROI within the outlined ROIs and included their ratios as well. Overall, quantitative parameters were more accurate in predicting the malignancy of a lesion compared with semi-quantitative and qualitative variables. This might be attributed to the fact that these parameters are more closely related to the pathophysiologic changes in a malignant tumor. The pharmacokinetic model used for the quantitative analysis in this study was first described by Tofts in 1999 as the standard two-compartmental model that includes plasma and the extravascular extracellular space (Tofts et al., 1999). The transport of gadolinium from vasculature to tissue interstitium corresponds to the blood flow and the permeability properties of the tissue that are reflected in the K_trans_ coefficient. K_ep_ represents the reverse transport of gadolinium into the vasculature. Neoangiogenesis is the physiological basis for this model, in which many small leaky vessels develop around the tumor to provide blood and nutrients for the increased demand of the fast-growing tissue. The characteristics of micro-vessels differ among benign and malignant lesions and so do the behavior of gadolinium which is measured by the pharmacokinetic factors.

The calculated AUC of ROC curves drawn for parameters measured for lROIs were the highest in the ratios of K_trans_ (tumor)/K_trans_ (myometrium), AUC-60 (tumor)/AUC-60 (myometrium) and Vb (tumor)/Vb(myometrium). The highest AUCs of parameters assessed on high signal points were found in the ratios of K_ep _(tumor)/ K_ep_ (myometrium), K_trans_ (tumor)/K_trans _(myometrium), V_b_(tumor)/V_b_ (myometrium) and AUC-60(tumor)/AUC-60(myometrium). 

Among these, the K_ep_ (tumor)/K_ep_ (myometrium) ratio measured from an sROI was found to be the best parameter for differentiating a malignant lesion from a benign mass with a sensitivity of 100%, specificity of 92.3%, PPV of 91.4%, and NPV of 100%. In comparison with the figures calculated for the qualitative method of the kinetic curves (sensitivity=71.4%, specificity=100%, PPV=100% and NPV=80.9%), this parameter seems to be considerably more accurate and reliable for diagnosing malignant lesions. The other mentioned ratios with AUCs of greater than 0.9 can also be used as diagnostic measures as well.

Based on the results of the present survey, it seems that outlining a large ROI throughout the whole solid part of the tumor (lROI) and using the mean measurements of the total area are not needed and that they do not add any value to our analyses. In contrast, it seems that using a small ROI (sROI) within the lesion would result in a higher accuracy for differentiating malignant lesions from the benign ones.

Aiming to find simpler diagnostic tools for ovarian cancer, the UK collaborative Trial of Ovarian Cancer Screening (UKCTOCS) compared the diagnostic characteristics of annual CA-125 screening plus trans-vaginal ultrasound assessment (multimodal screening) with annual screening by trans-vaginal ultrasound alone (Menon et al., 2009). The results of their robust study on 202,638 women aged 50 to 74 recruited from 13 centers in National Health Service Trusts in England, Wales, and Northern Ireland revealed a higher diagnostic value for multimodal screening method. According to their findings, for all primary ovarian and tubal cancers the multimodal screening method had a sensitivity of 89.4%, a specificity of 99.8%, and a PPV of 43.3% while these figures for trans-vaginal ultrasound alone were 84.9%, 98.2%, and 5.3%, respectively. For primary invasive epithelial ovarian and tubal cancers, the mentioned values for multimodal screening were 89.5%, 99.8%, and 35.1%, and for the ultrasound alone were 75.0%, 98.2%, and 2.8%, respectively. As can be seen there are multiple semi-quantitative and quantitative parameters in the DCE-MRI analysis that provide superior diagnostic values to the evaluated screening methods by UKCTOCS; however, the methods we evaluated should be conducted on a large sample population to yield more accurate estimates of their diagnostic characteristics. Moreover, the costs and availability of a diagnostic test is also an important factor that should be considered in choosing the best method and in this regard, multimodal screening and trans-vaginal ultrasound assessment seem to be better options. Nevertheless, to acquire concrete and reliable information on this matter, a comparison on the cost-effectiveness of these diagnostic tools is recommended.

Not including the morphological characteristics of the tumors, was one of the limitations of this study. Accordingly, we could not compare the classical morphological criteria of malignancy with the assessed parameters. Another major shortcoming of the present survey was categorizing the patients into two groups of benign or malignant lesions and not separately analyzing borderline tumors. Therefore, in order to make a decision regarding the application of these parameters as a part of evaluations for ovarian masses, further investigations are required including larger sample populations. 

In conclusion, our study showed that further evaluation is not needed if the lesion has TIC Type I or Type III in qualitative DCE-MRI. Complementary quantitative analysis is only recommended for the adnexal masses with Type II TICs. Semi-quantitative and quantitative DCE-MRI parameters measured from either an lROI or sROI can be used for differentiating malignant ovarian lesions from benign masses. However, the accuracy of quantitative parameters was found to be higher than that of semi-quantitative variables, particularly when measured from sROI rather than lROI. The K_ep_ (tumor)/K_ep_ (myometrium) ratio measured from one sROI within the outlined ROI was found to be the most accurate discriminator (sensitivity=100%, specificity=92.3%, PPV=91.4% and NPV=100%).
